# Decoys Selection in Benchmarking Datasets: Overview and Perspectives

**DOI:** 10.3389/fphar.2018.00011

**Published:** 2018-01-24

**Authors:** Manon Réau, Florent Langenfeld, Jean-François Zagury, Nathalie Lagarde, Matthieu Montes

**Affiliations:** Laboratoire GBA, EA4627, Conservatoire National des Arts et Métiers, Paris, France

**Keywords:** virtual screening, benchmarking databases, benchmarking, decoy, structure-based drug design, ligand-based drug design

## Abstract

Virtual Screening (VS) is designed to prospectively help identifying potential hits, i.e., compounds capable of interacting with a given target and potentially modulate its activity, out of large compound collections. Among the variety of methodologies, it is crucial to select the protocol that is the most adapted to the query/target system under study and that yields the most reliable output. To this aim, the performance of VS methods is commonly evaluated and compared by computing their ability to retrieve active compounds in benchmarking datasets. The benchmarking datasets contain a subset of known active compounds together with a subset of decoys, i.e., assumed non-active molecules. The composition of both the active and the decoy compounds subsets is critical to limit the biases in the evaluation of the VS methods. In this review, we focus on the selection of decoy compounds that has considerably changed over the years, from randomly selected compounds to highly customized or experimentally validated negative compounds. We first outline the evolution of decoys selection in benchmarking databases as well as current benchmarking databases that tend to minimize the introduction of biases, and secondly, we propose recommendations for the selection and the design of benchmarking datasets.

## Introduction

Computer-aided drug design (CADD) is now a commonly integrated tool in drug discovery processes (Sliwoski et al., 2014). It represents a way to predict ligands bioactivity *in silico*, and help focusing the drug discovery efforts on a limited number of promising compounds, saving both time and money in this very competitive field. Among these computational methods, Virtual Screening (VS) is designed to prospectively help identifying potential hits, i.e., compounds able to interact with the target and to modulate its activity, out of large compound collections (Tanrikulu et al., 2013). VS approaches can be Ligand-Based (LBVS) when they rely only on the structure/properties of known active compounds to retrieve promising molecules from compound collections (using similarity search, QSAR or 2D/3D pharmacophore, etc.), or Structure-Based (SBVS) if the structural information of the target is used (like in molecular docking studies).

The evaluation of VS methods is crucial prior to large library prospective screening to select the appropriate methodology, and subsequently generate reliable outcome on real-life project. Thus, software and workflows must be thoroughly evaluated retrospectively using benchmarking datasets. Such datasets are composed of known active data together with inactive compounds referred to as “decoys” (Irwin, 2008). Ideally, both active and inactive compounds should be selected on the basis of experimental data. However, the documentation on inactive data is scarce, and putative inactive compounds are generally used instead. Among the common metrics used to estimate the performance of VS methods we find receiver operating characteristics (ROC) curves, the area under the ROC curve (ROC AUC) (Triballeau et al., 2005), Enrichment Curves (EC), Enrichment Factors (EF) and predictiveness curves (Empereur-mot et al., 2015). While conceptually different, they all share the same objective: assess the ability of a given method to identify active compounds as such, and discriminate them from the decoy compounds.

However, since the publication of the first benchmarking database in the early 2000s, the composition in both active and decoy compounds have been pointed out to crucially impact VS methods evaluation; several biases have been shown to incline VS assessment outcomes positively or negatively. The difference between the two chemical spaces defined by the active compounds on the one hand and the decoy compounds on the other hand may lead to artificial overestimation of the enrichment (Bissantz et al., 2000). On the contrary, the possible presence of active compounds in the decoy compounds set may introduce an artificial underestimation of the enrichment (Verdonk et al., 2004; Good and Oprea, 2008) since decoys are usually assumed to be inactive rather than proved to be true inactive compounds (i.e., confirmed inactive through experimental bioassays). New databases were designed to minimize those biases (Rohrer and Baumann, 2009; Vogel et al., 2011; Mysinger et al., 2012; Ibrahim et al., 2015a). Finally, many studies pointed out that the VS performance depends on the target and its structural properties (structural flexibility, binding site physicochemical properties, etc.; Cummings et al., 2005). Taking this into consideration, and despite the growing number of protein families represented in databases, decoy datasets generation tools were made publicly available in order to allow any scientist to fine-tune target-dependant and reliable benchmarking datasets (Mysinger et al., 2012; Ibrahim et al., 2015a).

In this review, we first present how the notion of decoy compounds evolved from randomly selected putative inactive compounds to rationally selected putative inactive compounds and finally true negative compounds. We develop the successive benchmarking datasets that were published in the literature and their basic to highly refined decoys selection workflows together with the resulting positive or negative biases due to their design. We then detail 5 benchmarking databases or decoy sets generator tools along with their detailed decoy compounds selection that represent the current state-of-the-art as of 2017: their respective composition tend to minimize such biases. Finally, we propose recommendations to select minimally biased benchmarking datasets containing putative inactive compounds as decoy compounds and introduce guidelines to design true inactive compounds containing databases.

## The history of decoys selection

### Randomly selected decoys

The first use of a benchmarking database to evaluate virtual screening tools dates back to 2000, with the pioneering work of Bissantz et al. (2000). The objective of their study was to evaluate the ligands enrichment, i.e., the ability of docking programs to associate active compounds with the best scores within a compound collection. Three docking programs [Dock (Kuntz et al., 1982), FlexX (Rarey et al., 1996), Gold (Jones et al., 1997)] combined with 7 scoring functions [ChemScore (Eldridge et al., 1997), Dock, FlexX, Fresno (Rognan et al., 1999), Gld, Pmf (Muegge and Martin, 1999), Score (Wang et al., 1998)] were evaluated on two different target proteins: Thymidine Kinase (TK) and the ligand binding domain of the Estrogen Receptor α subtype (ER α).

For each target, a dataset containing 10 known ligands and 990 molecules assumed to be inactive (decoy compounds) was created. The decoy compounds were selected following a two-step scheme: (1) the Advanced Chemical Directory (ACD v.2000-1, Molecular Design Limited, San Leandro) was filtered to eliminate undesired compounds (chemical reagents, inorganic compounds and molecules with unsuitable molecular weights), (2) 990 molecules were randomly selected out of the filtered dataset. The datasets were used to evaluate and compare several docking and scoring schemes. The authors eventually recommended a calibration of docking/consensus scoring schemes on reduced data sets prior to large dataset screens. Later on, Bissantz et al. (2003) applied the same protocol to three human GPCRs to investigate whether their homology models were suitable for virtual screening experiments.

A growing interest for virtual screening benchmarking databases soon emerged from the community (Kellenberger et al., 2004; Brozell et al., 2012; Neves et al., 2012; Repasky et al., 2012; Spitzer and Jain, 2012). New databases were designed with an increasing complexity in the decoys selection methodologies (see section Benchmarking Databases). Nowadays, benchmarking databases are widely used to evaluate various VS tools (Kellenberger et al., 2004; Warren et al., 2006; McGaughey et al., 2007; von Korff et al., 2009; Braga and Andrade, 2013; Ibrahim et al., 2015a; Pei et al., 2015) and to support the identification of hit/lead compounds using LBVS and SBVS (Allen et al., 2015; Ruggeri et al., 2015).

### Integration of physicochemical filters to the decoy compounds selection

In the early 2000s, Diller's group incorporated filters in the decoys selection to ensure that the discrimination they observed was not solely based on the size of the decoy compounds (Diller and Li, 2003). In addition to the 1,000 kinases inhibitors they retrieved from the literature for 6 kinases (EGFr, VEGFr1, PDGFrβ, FGFr1, SRC, and p38), 32,000 compounds were randomly selected from a filtered version of the MDL Drug Data Report (MDDR). The filters were designed to select decoy compounds displaying similar polarity and molecular weight. Similarly, in 2003, a benchmarking database derived from the MDDR was constructed by McGovern et al. (McGovern and Shoichet, 2003). Compounds with unwanted functional groups were removed, leading to 95,000 compounds. The targets of the MDDR for which at least 20 known ligands were available constituted a target dataset (CA II, MMP-3, NEP, PDF, and XO). The remaining compounds were used as decoy compounds. The addition of rational filters was a considerable step forward in the improvement of decoys selection, but due to the commercial licensing of the MDDR, its use was limited (http://www.akosgmbh.de/accelrys/databases/mddr.htm^1^).

The first benchmarking databases were composed as follows: (1) true active compounds consisted in known ligands extracted from the literature while (2) decoy compounds consisted in putative inactive compounds randomly selected from large databases possibly filtered to be compliant to specific criteria (drug likeness, molecular weight, topological polar surface area…). Since the decoy compounds were pseudo-randomly selected, they were assumed to be inactive on the defined targets.

Despite the use of the MDDR and the filtering of the decoy compounds, these benchmarking databases displayed a major drawback: the significant differences occurring between the physicochemical properties of the active compounds and decoy compounds led to obvious discrimination and then artificially good enrichments (Verdonk et al., 2004; Huang et al., 2006).

In 2006, Irwin et al. proposed that the decoy compounds should be similar to the known ligands regarding their physicochemical properties to reduce the introduction of bias while being structurally dissimilar to the known ligands to reduce their probability to be active on the defined target. Following these recommendations, they created the DUD database (Huang et al., 2006) that was immediately considered as the gold standard for the evaluation of VS methods.

The DUD database is composed of 2,950 ligands and 95,326 decoys for a total of 40 proteins from 6 classes (nuclear hormone receptors, kinases, serine proteases, metalloenzymes, folate enzymes and others). The decoy compounds were extracted from the drug-like subset of the ZINC database (Irwin and Shoichet, 2005). The 2D-similarity between known ligands and decoy compounds was computed by calculating the Tanimoto distance based on the CACTVS type 2 substructure keys and 5 physicochemical properties. For each active compound, the 36 molecules sharing the most similar properties while being topologically dissimilar (Tanimoto < 0.9) were conserved. The evaluation of the performance of DOCK (Meng et al., 1992; Wei et al., 2002; Lorber and Shoichet, 2005; Huang et al., 2006) confirmed that uncorrected databases such as the MDDR led to over-optimistic enrichments compared to corrected databases such as the DUD.

### Benchmarking database biases

Despite the precautions taken to build the DUD database, several remaining biases have been reported in the literature.

The “analogous bias” (Good and Oprea, 2008) lies in the limited chemical space of active compounds that is restricted to the chemical series that have been explored and referenced in databases. The discrimination of the active compounds from decoy compounds can be simplified since the decoy sets would display a larger structural variability that could induce an overestimation of the performance of VS methods. The lack of diversity in the structures of known active compounds limits the training and evaluation of LBVS methods to perform scaffold-hopping, i.e., the identification of active hit compounds that structurally differ from reference molecules while retaining similar activity.

The “complexity bias” (Stumpfe and Bajorath, 2011) or “artificial enrichment bias”: active compounds and decoy compounds often display differences in their respective structural complexity since active compounds are often optimized compounds extracted from large series in the scientific and patent literature, which is not necessarily the case for the structures of pseudo-randomly selected decoy compounds.

The “false negative bias” (Vogel et al., 2011; Bauer et al., 2013) lies in the presence of active compounds in the decoy set. Unlike the analogous and complexity biases, it induces an underestimation of the performance of the VS methods that could be particularly dramatic for the evaluation of LBVS methods (Irwin, 2008).

The need for less biased benchmarking databases to objectively evaluate VS methods favored the emergence of new strategies to eradicate or at least minimize those biases. Two decoys selection strategies arose from benchmarking databases improvement attempts: (1) the use of highly refined decoys selection strategies and (2) the integration of true negative compounds in the decoy set.

### Highly refined putative inactive compounds selection

The reported biases pointed out that the composition of both active compounds and decoy compounds sets has a huge impact on the evaluation of the performance of VS methods (Verdonk et al., 2004; Good and Oprea, 2008). Therefore, particular efforts were performed in the selection strategies for active compounds and decoy compounds.

To address analogous bias, a strategy consists in modifying the receiver operating characteristics (ROC) curves (i.e., the fraction of actives among the top fraction *x* of the data set) (Triballeau et al., 2005) by weighting the rank of each active compound with the size of its corresponding lead series (Clark and Webster-Clark, 2008). This allows an equal contribution of each active chemotype to the ROC curve (rather than each active compound). Another widely used method is to fine-tune the active compounds dataset prior to screen to ensure an intrinsic structural diversity. To this aim, the MUV datasets (Rohrer and Baumann, 2009) were designed using the Kennard Jones algorithm to obtain an optimal spread of the active compounds in the decoy compounds chemical space while ensuring a balance between the active compounds self-similarity and separation from the decoy compounds. Despite these observations, the most used strategy in the literature still consists in clustering ligands based on 2D descriptors and retain only cluster representatives in the final dataset (Good and Oprea, 2008; Mysinger et al., 2012; Bauer et al., 2013).

To reduce artificial enrichment, efforts were made to match as much as possible the physicochemical properties of the decoys to the physicochemical properties of the active compounds. To this aim, the Maximum Unbiased Validation database (MUV) (Rohrer and Baumann, 2009) was designed to ensure embedding of active compounds in the decoy compounds chemical space based on an embedding confidence distance cut-off calibrated on multiple drug-like compounds banks' chemical space. Active compounds that were poorly embedded in the decoy set were discarded. A way to ensure the availability of potential decoy compounds for any ligand is to generate decoys that ignore synthetic feasibility (Wallach and Lilien, 2011). Other databases select decoys that match active compounds in a multiple physicochemical properties space. The DEKOIS 2.0 (Ibrahim et al., 2015a) proposed a workflow that used 8 physicochemical properties while the DUD-E added net charge to the 5 physicochemical properties already considered in the original DUD.

To address the risk of including false negatives in the decoy set, a common strategy is to select decoy compounds topologically different to any active compound. For this purpose, Bauer et al. introduced the LADS score to guide decoys selection (Vogel et al., 2011). In the DUD-E, potential false decoys are avoided by applying a stringent FCFP_6 fingerprints Tanimoto-based filter. It is important to note that since the evaluation of LBVS methods requires that decoy compounds should not be discriminated using basic 2D-based similarity tools, the use of 2D-based dissimilarity filters to avoid false negatives in the decoy set makes the concerned databases inappropriate for the evaluation of the performance of LBVS methods. Therefore, Xia et al. developed a method to select adequate decoys for both SBVS and LBVS (Xia et al., 2014) by favoring physicochemical similarity as well as topological similarity between active compounds and decoy compounds that passed a primary topological dissimilarity filter.

With these improvements, the notion of decoys remained the same—putative inactive compounds—but their selection critically evolved. Ever since, the main progress achieved in the literature lies in the diversification of the protein targets represented in benchmarking databases. The growing need for datasets dedicated to a given target led to (1) an increasing diversity of targets in benchmarking databases [the DUD-E (Mysinger et al., 2012) contains datasets against 102 targets while the previous DUD (Huang et al., 2006) contained datasets only for 40 targets] and (2) highly specialized benchmarking databases focused on a particular class of targets. Such specialized datasets exist for GPCRs [GPCR ligand library (GLL)/Decoy Database (GDD) (Gatica and Cavasotto, 2012)], histone deacetylases [maximal unbiased benchmarking data sets for HDACs—MUBD-HDACs (Xia et al., 2015)], or nuclear receptors [NRLiSt BDB (Lagarde et al., 2014a)]. As a notice, DUD-E or DecoyFinder (Cereto-Massagué et al., 2012) offer automated decoy set generation tools based on the properties of active compounds, enabling the community to easily design and tune their own dataset for a particular target.

### Toward true negative compounds

A common issue about decoys is the lack of data regarding their potential bioactivity against the target. Most methods assume that the absence of data means an absence of activity, which may lead to include unknown active ligands into a decoy set. To eliminate such false negatives from decoy sets, one solution is to use referenced true negative compounds that can be either true inactive or compounds displaying an undesirable activity.

True inactive compounds, i.e., compounds that displayed no experimental binding affinity against the target of interest, can be used to identify binders. Inactive data is made available in several public activity and/or affinity annotated compound repositories and high throughput screening (HTS) initiatives such as: ChEMBL (Bento et al., 2014), Drugbank (Wishart et al., 2008) that provides annotations for approved drugs; PDBBind (Wang et al., 2004, 2005), Binding MOAD (Benson et al., 2008) and AffinDB (Block et al., 2006) that contain binding affinity data for protein–ligand complexes available in the Protein Data Bank (PDB) (Berman et al., 2000); PDSP Ki database (Roth et al., 2000) that stores screening data from the National Institute of Mental Health's Psychoactive Drug Screening Program; BRENDA (Placzek et al., 2017) that provides binding constants for enzymes; IUPHAR (Southan et al., 2016) that contains binding information for receptors and ion channels; GLIDA (Okuno et al., 2006) and GPCRDB (Munk et al., 2016) that contains binding data for G-protein-coupled receptors; D3R datasets (Drug Design Data Resource^2^) that have been provided by pharmaceutical companies and academia and contain affinity data for 7 proteins together with inactive compounds; ToxCast™/Tox21 (Kavlock et al., 2012) and PCBioAssay (Wang et al., 2017) that provide HTS data for various targets.

As an example, the DUD-Enhanced (Mysinger et al., 2012) (DUD-E) integrates some experimentally validated inactive compounds extracted from ChEMBL in the decoy set in addition to putative inactive compounds: an arbitrary 1 μM cutoff is used to classify ligands in the active set while molecules with no measurable activity at 30 μM or higher concentration were classified into the decoy set. Similarly, the Maximum Unbiased Validation (MUV) (Rohrer and Baumann, 2009) datasets are composed of both active and inactive compounds collected from the PubChem BioAssay annotated database.

Unwanted compounds, i.e., compounds that display unwanted activity or binding, can also be used as negatives. For instance, a recent study used ligands of the NRLiSt BDB (Lagarde et al., 2014a) either as active compounds or decoy compounds, depending on their activity for each nuclear receptor; antagonist (or agonist) ligands of a given nuclear receptor were used as decoys to evaluate agonistic (or antagonistic) pharmacophores (Lagarde et al., 2016, 2017). This strategy has shown successful results in the past: Guasch et al. (2012) focused on PPAR γ partial agonists to avoid side effects accompanying full receptor activation and built an anti-pharmacophore model with known full agonist compounds to remove all potential full agonist compounds from their initial set of 89,165 natural products and natural product derivatives. The authors screened the remaining compounds on a partial agonist pharmacophore model and identified 135 compounds as potential PPARγ partial agonists with good ADME properties among which 8 compounds with new chemical scaffolds for PPARγ partial agonistic activity. After biological tests, 5 compounds were confirmed to be PPAR γ partial agonists.

## Benchmarking databases

**Table:** 

**DB name**	**Year**	**Download address**	**Origin of the ligands**	**Origin of the decoys**	**No. of targets / No. of classes**	**Decoy compounds selection**	**Remarks**
Rognan's decoy set (Bissantz et al., 2000)	2000	http://bioinfo-pharma.u-strasbg.fr/labwebsite/download.html	Literature	ACD	2/2	Random selection	Design of decoy sets to evaluate the performance of 3 docking programs and 7 scoring functions
Shoichet's decoy set (McGovern and Shoichet, 2003)	2003	–	MDDR	MDDR	9/4	Remove compounds with unwanted functional groups	Compare VS performance depending on the binding site definition (apo, holo or homology modeled structures)
Li's decoy set (Diller and Li, 2003)	2003	–	Literature	MDDR	6/1	Fit polarity and MW to known kinases inhibitors	Compare decoys and ligands physicochemical properties to select decoys
Jain's decoy set (Jain and Nicholls, 2008)	2006	http://www.jainlab.org/downloads.html	PDBbind	ZINC “drug-like” and Rognan's decoy set	34/7	1,000 random molecules from the ZINC that comply to MW ≤ 500, logP ≤ 5, HBA ≤ 10, HBD ≤ 5 and RB ≤ 12 and Rognan's decoys with RB ≤ 15	Use of 5 physicochemical properties to match decoy sets to ligands sets
Directory of Useful Decoys (DUD) (Huang et al., 2006)	2006	http://dud.docking.org	Literature and PDBbind	ZINC “drug-like”	40/6	Decoys must be Lipinski-compliant. The selection is based on both the topologically dissimilarity to ligands and the fit of physicochemical properties	Largest decoy data set so far (40 proteins) and first attempt to select decoys topologically dissimilar decoys
DUD Clusters (Meyer, 2007)	2008	http://dud.docking.org/clusters/	DUD	–	40/6	–	DUD clusters more relevant for scaffold hopping
WOMBAT Datasets (Meyer, 2007)	2007	http://dud.docking.org/wombat/	WOMBAT	–	13/4	–	Design to decrease the analog bias on 13 of the 40 DUD targets, enrich DUD active data sets with compounds from WOMBAT database
Maximum Unbiased Validation (MUV) (Rohrer and Baumann, 2009)	2009	–	PubChem	PubChem	18/7	Two functions measure the active-active and decoy-active distances using 2D chemical descriptors. Actives with the maximum spread within the active set were chosen and decoys with similar spatial distribution were selected	Ligands and decoys are from biologically actives and inactive compounds, i.e., are true actives and inactives, respectively
DUD LIB	2009	http://dud.docking.org/jahn/	DUD-cluster	DUD	13/4	Subset of the DUD database, with more stringent criteria on MW (≤450) and AlogP (≤4,5), and a minimal number of chemotypes	Initially designed for “scaffold-hopping” studies
Charge Matched DUD	2010	http://dud.docking.org/charge-matched/	DUD	ZINC	40/6	Apply a net charge property match on DUD datasets	
REPROVIS-DB	2011	–	Literature	Literature	–	Extracted from previous successful studies	Designed for LBVS only
Virtual Decoy sets (VDS) (Wallach and Lilien, 2011)	2011	http://compbio.cs.toronto.edu/VDS	DUD	ZINC	40/6	Same as DUD, but does not consider synthetic feasibility	Purely virtual decoys, availability is not considered
DEKOIS (Vogel et al., 2011)	2011	http://dekois.com/dekois_orig.html	BindingDB	ZINC	40/6	Class decoys and ligands into “cells” based on 6 physicochemical properties and select the closest decoys based on (1) a weighted physicochemical similarity and (2) a LADS score based on functional fingerprints similarity elaborated from the active set	Original treatment of the physicochemical similarity, and introduce the concept of *Latent Active in Decoy set*, i.e., false false positives
GPCR Ligand (GLL)/Decoys Database (GDD) (Xia et al., 2014)	2012	http://cavasotto-lab.net/Databases/GDD/	GLIDA and PDB structures and Vilar et al., 2010	ZINC	147/1	Physico-chemical properties fit and topological dissimilarity filter. Final selection based on MW	First extensive database targeting a specific protein family
Decoy Finder (Cereto-Massagué et al., 2012)	2012	http://urvnutrigenomica-ctns.github.io/DecoyFinder/	User	User	–	Same as DUD	Graphical tool to generate decoy data sets with adaptable thresholds for physicochemical properties
DUD Enhanced (DUD-E) (Mysinger et al., 2012)	2012	http://dud.docking.org/r2/	CHEMBL	ZINC	102/8	Physico-chemical properties fit along with a topological dissimilarity filter. Random selection of decoys is then applied	Largest database so far (1,420,433 decoys and 66,695 actives)
DEKOIS 2.0 (Ibrahim et al., 2015a)	2013	http://www.dekois.com	BindingDB	ZINC	81/11	Same as DEKOIS with 3 additional physicochemical properties (nFC, nPC, Ar), a PAINS filter and an improved, weighted LADS score	
NRLiSt BDB (Lagarde et al., 2014a)	2014	http://nrlist.drugdesign.fr	CHEMBL	ZINC and DUD-E decoys generator	27/1	Use the DUD-E decoy generation tool	Ligands can be either agonists or antagonists (other actives are removed), depending on the purpose of the study
MUBD-HDACs (Xia et al., 2015)	2015	–	CHEMBL and literature	ZINC	14/1	Select decoys based a weighted physicochemical similarity (6 physicochemical properties are considered), and ensure a random spatial distribution of the decoys (i.e., decoys should be as distant to the other actives as a reference ligand)	Applicable both to SBVS and LBVS strategies, uses ligands with proved bioactivity

## Selected databases

### Maximum unbiased validation (MUV)

The MUV was designed to propose unbiased datasets in regard to both artificial enrichment and analogous bias by proposing a new approach gleaned from spatial statistics (Rohrer and Baumann, 2009). The authors ensured homogeneity in actives-actives similarity and actives-decoys dispersion in order to reach a random-like distribution of active compounds and decoy compounds in a physicochemical descriptors chemical space. This implies that the molecular properties contained no information about the bioactivities of active and decoy compounds. Datasets were designed for 18 targets with a total of 30 actives and 15,000 decoys for each target.

#### Initial compounds database

Potential active and decoy compounds were extracted from HTS experiments available in PCBioAssay (June 2008) (PubChem BioAssay^3^). In these assays, a primary screen was performed in a large number of compounds (>50,000) and was followed by a low throughput confirmatory screen. Compounds with an experimental EC50 in the confirmatory screen were selected as potential active compounds while inactive compounds from the primary screen were selected as potential decoys.

#### Actives selection

**Figure 1:**
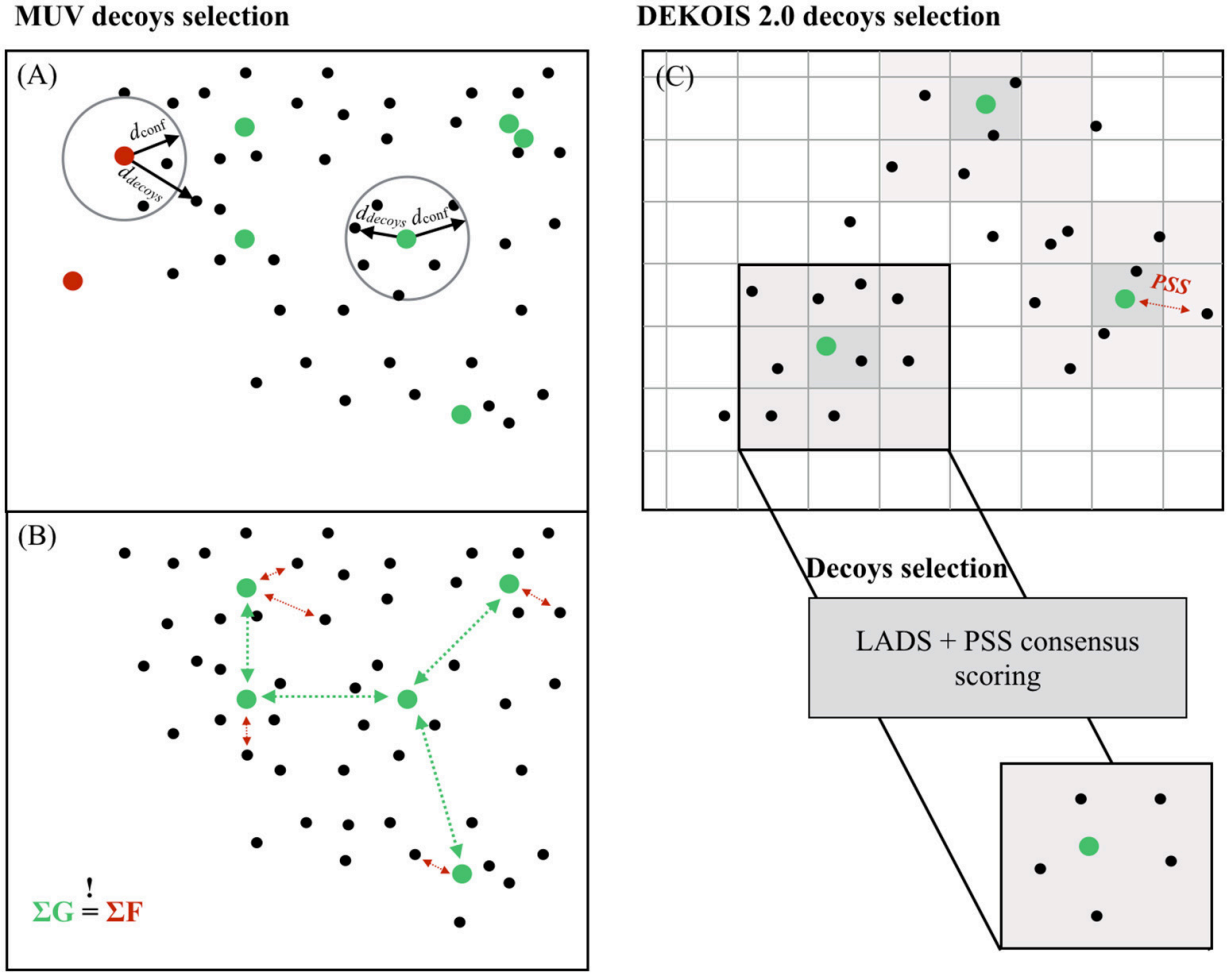
Decoys selection in MUV and DEKOIS 2.0. **(A)** For each active of the MUV, a distance to the 500th nearest neighbor from 100 random samples from multiple drug-like compounds collections was computed. The 90th percentile was recorded as the confidence distance for a good embedding (d_*conf*_). Active compounds were accepted only if the 500th nearest neighbor from the decoy compounds (d_*decoys*_) set was within the d_*conf*_. **(B)** Selected active compounds datasets from the MUV were adjusted to the same level of spread (ΣG ≈ constant), and decoy compounds sets were, in turn, adjusted to this level of spread (ΣF ≈ ΣG). **(C)** The chemical space of both active and decoy compounds was divided into cells characterized by a set of 8 physicochemical properties. Each user-provided compound is associated with its property matching cell, and 1,500 decoys are selected from the same cell, or direct neighboring cells if the parent cell is not populated enough.

A two-step process was applied to rationally select final active compounds for the MUV data sets. (1) Potential active compounds were filtered to eliminate artifacts caused by organic chemicals aggregation in aqueous buffers (“Hill slope filter”), as well as off-targets, cytotoxic effects or interference with optical detection methods [“frequency of hits filter” and “autofluorescence (Simeonov et al., 2008) and luciferase inhibition (Auld et al., 2008) filters”]. (2) A “chemical space embedding filter” was applied to ensure that actives located in regions of the chemical space devoid of decoys were eliminated from the dataset (Figure 1). Subsets of 30 actives with the maximum spread per target were generated using a Kennard-Jones algorithm. Selected active compounds were exchanged with remaining potential active compounds until all datasets were adjusted to a common level of spread.

#### Decoys selection

To carefully match active and decoys physicochemical properties, Rohrer et al. proposed that the level of self-similarity within the active compounds set [measured using the “nearest neighbor function” *G*(*t*)] should be equal to the degree of separation between the active compounds set and the decoy compounds set [evaluated with the “empty space function” *F*(*t*)] (Figure 1). Following guideline, the data clumping should be null, ensuring a random-like distribution of decoy and active compounds in the overall chemical space. The distances were computed based on 1D molecular properties (counts of all atoms, heavy atoms, boron, bromine, carbon, chlorine, fluorine, iodine, nitrogen, oxygen, phosphorus, and sulfur atoms in each molecule as well as the number of H-bond acceptors, H-bond donors, the logP, the number of chiral centers, and the number of ring systems). The level of separation between the decoy compounds and the active compounds was adjusted to the same level of spread so that the data clumping is null. In total, 500 decoys were selected per selected active, resulting in 15,000 decoys per dataset.

The minimization of analog bias and artificial enrichment makes the MUV datasets fitted for LBVS. The availability of structures in the PDB (2008) for seven of the MUV targets makes it suitable for SBVS as well (Löwer et al., 2011). Thus, the MUV constituted the first dataset that enabled comparative evaluations of SB and LBVS methods and protocols.

### Demanding evaluation kits for objective *in silico* screening (DEKOIS)

In 2011, Vogel et al. proposed a new generator of decoy compounds sets called *Demanding Evaluation Kits for Objective In Silico Screening* (DEKOIS) (Vogel et al., 2011). The authors designed their tool to avoid the introduction of well-known and described biases into the decoy sets, i.e., analog bias and artificial enrichment. A first step in their workflow is subsequently to closely match physicochemical properties of both ligand and decoys to limit the analog bias. Then, to deal with the risk of including false negative compounds in the decoy compounds set, a new concept is applied to the decoys selection process: the *latent actives in the decoy set* (LADS). Finally, the structural diversity of the active and decoy compounds structures into the sets is evaluated and maximized, and the embedding of the actives into the decoys chemical is assessed. The whole workflow was further improved in 2013 to produce the current version of this tool, DEKOIS 2.0 (Bauer et al., 2013), and 81 ready-to-use (active and decoys) benchmarking datasets for 11 target classes are currently available through the DEKOIS website (www.dekois.com/, accessed 10/23/2017).

#### Initial compounds database

Decoy compounds from the DEKOIS 2.0 benchmarking datasets are selected from a subset of the ZINC database of 15 million molecules. Eight physicochemical properties are evaluated: molecular weight, octanol–water partition coefficient, hydrogen bonds acceptor and/or donor, number of rotatable bonds, positive and negative charges, and the number of aromatic rings. For each physicochemical property, bins are defined, and all possible combinations of bins are used to split the database compounds into cells. The initial bins are defined so that each bin is equally populated, and each final cell is characterized by a set of 8 physicochemical properties. Each user-provided active compound is associated with the closest cell (in terms of physicochemical properties), and 1,500 decoys are randomly preselected from this parent cell, or from the direct neighbor cells if the parent cell is not populated enough to provide 1,500 decoy compounds (Figure 1).

#### Decoys selection

The two criteria for the refinement steps are the structural diversity and the low rate of *latent active in decoy set* (LADS). A physicochemical similarity score (PSS) and a LADS score are computed, normalized and combined to select the final 30 decoys associated with each active ligand:

1. The PSS score is the arithmetic mean of the normalized distance between a decoy and the reference ligand, for each physicochemical property.
2. The avoidance of LADS relies on the fingerprints bit strings shared by the active compounds: the fingerprint bit strings of each preselected decoy compound is matched to the fingerprint bit strings of all active compounds using the following:
  $$\begin{array}{l} LADS\;score=\frac{\sum_{i=1}^{n}\left(N_{i\left(HeavyAtoms\right)}\cdot f_{i\left(FCFP_{6}fragment\right)}\right)}{N_{FCPC_{6}fragments}}, \end{array}$$
  with *n* the number of fingerprint bit strings shared by the decoy and the active set, *f*_*i*_ the frequency of fragment *i* in the active set, *N*_*i*_ the number of heavy atoms into fragment *i*, and *N* the total number of FCPC_6 fragments into the decoy.
  The weighting of the LADS score by the frequency of the bit string and the size of the corresponding fragment was added in the second version of DEKOIS (Bauer et al., 2013) to ensure that large bioactive substructures and substructures frequently found exert a greater influence on LADS score compared to smaller and rare functional groups.
3. The LADS and PSS scores are normalized and combined into a consensus score to sort decoy compounds. The subsequently best 100 decoys are selected. Finally, the fingerprints are used to select the 30 most dissimilar decoys for each active.

Using this enhanced protocol, Bauer et al. showed an improvement of the “deviation from optimal embedding score” (DOE score) (Vogel et al., 2011; Bauer et al., 2013) for DEKOIS 2.0 compared to DEKOIS, and found a good (<0.2) DOE score for 89% out of the 81 targets considered.

### Dud-enhanced (DUD-E)

**Figure 2:**
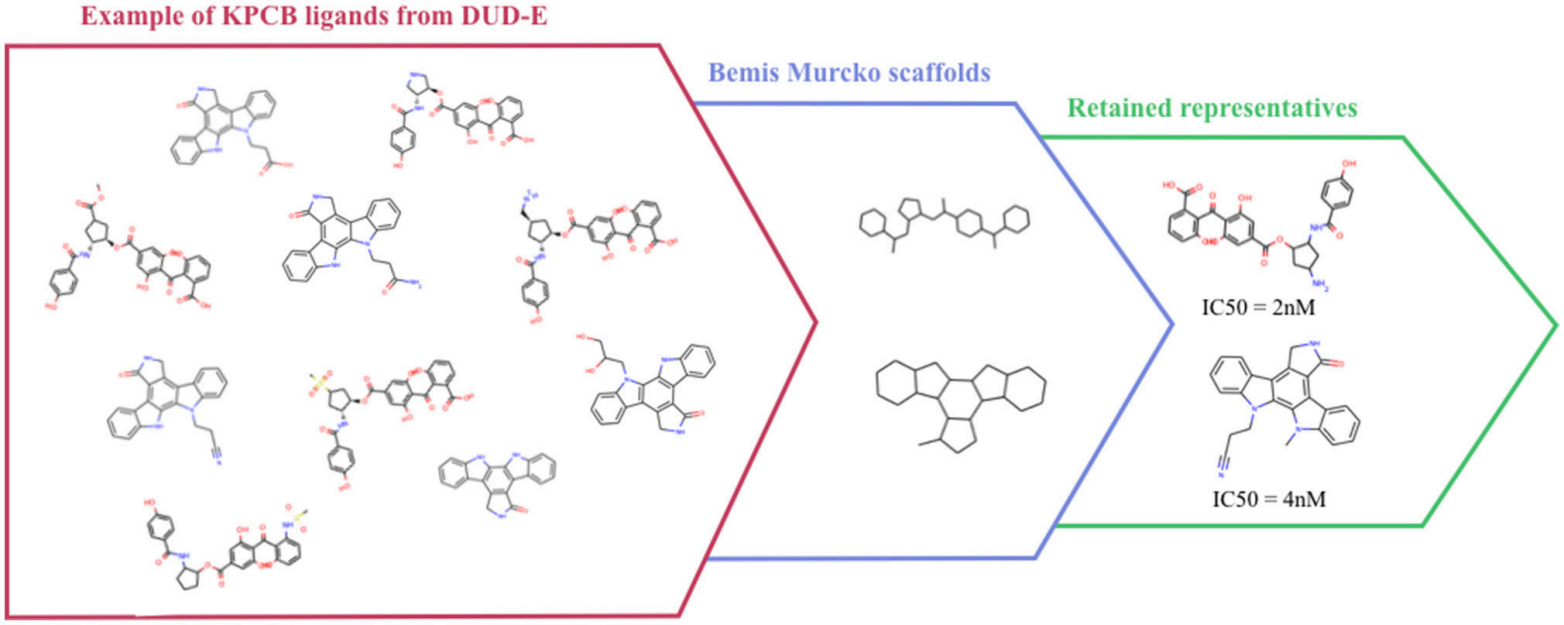
Example of Bemis-Murcko atomic frameworks clustering of Protein kinase C beta type (KPCB) ligands from the DUD-E.

Despite the extensive use of the DUD, several studies pointed out that some scaffolds were over-represented in the active sets, that the charge was not considered in property-matching for ligand selection, and that true ligands could be found in the decoy sets (Good and Oprea, 2008; Hawkins et al., 2008; Irwin, 2008; Mysinger and Shoichet, 2010). Shoichet et al. proposed the DUD-E (DUD-Enhanced) to address these weaknesses in both the active and the decoy sets design in the DUD, and extended the number of represented protein families in the database. The DUD-E contains 102 proteins that span diverse target classes. To address analogous bias, ligands were clustered by their Bemis-Murcko atomic frameworks (Bemis and Murcko, 1996) (Figure 2), and a topological dissimilarity filter was applied to avoid active compounds in the decoy sets.

#### Initial compounds database

Active compounds assigned to each target of the DUD-E were collected from the ChEMBL09 database if their activity/affinity (Ki, Kd, IC50, EC50, or associated logP) was ≤1μM (Gaulton et al., 2012). Additionally, 9,219 experimental decoys displaying no measurable affinity up to 30 μM were included in the decoy sets.

#### Active set preparation

Active compounds were clustered based on their Bemis-Murcko atomic frameworks. When more than 100 frameworks were represented, the highest energy ligand from each cluster is considered, while when less than 100 frameworks are represented, the numbered of considered ligands was raised to obtain more than 100 molecules. Even if this selection protocol could have been optimized for sets with low frameworks diversity, it ensures sufficient diversity and quantity of compounds for the other sets.

#### Decoys selection

The decoy compounds were extracted from the ZINC database (Irwin and Shoichet, 2005) and selected by narrowing or widening windows around 6 physicochemical properties: molecular weight, octanol-water partition coefficient, rotatable bonds, hydrogen bonds acceptors, hydrogen bonds donors, and the net charge. To avoid active compounds in the decoy sets, a topological dissimilarity filter was applied. Molecules were sorted according to their Tanimoto distance to any ligands using CACTVS fingerprints, and the 25% most dissimilar decoy molecules were retained. Finally, up to 50 decoys were randomly selected for each ligand and pooled with the 9,219 experimental decoys.

An automated tool was made available online to generate decoys from user-supplied ligands using the same protocol (http://decoys.docking.org). The possibility to generate decoy sets for any target has been revealed successful and is now widely used by the scientific community (Lacroix et al., 2016; Nunes et al., 2016; Allen et al., 2017; Meirson et al., 2017).

Despite the success of the DUD-E, some weaknesses should be corrected in the DUD-E benchmarking database. The 102 targets are defined as a UniProt gene prefix (such as DRD3) and not a full gene_species (such as DRD3_HUMAN or P35462), which can bias the actives selection when the binding site composition differs between species. Additionally, only one single structure was considered for each protein while many docking studies pointed out that the structure selection is crucial for screening and docking, particularly for proteins that accommodate ligands with different binding modes (May and Zacharias, 2005; Ben Nasr et al., 2013; Lionta et al., 2014). A recent study has shown that the ligand pharmacological profile should be considered for both the active set design and the structure selection (Lagarde et al., 2017). For instance, nuclear receptors (NR) can be inhibited by antagonists or activated by agonists that differ in their structure and properties: agonists should be considered in the active set if the screening is performed on an agonist-bound structure while antagonists should be used in the active set if the screening is performed on an antagonist-bound structure.

### Nuclear receptors ligands and structures benchmarking database (NRLiSt BDB)

The NRLiSt BDB (Nuclear Receptors Ligands and Structures Benchmarking DataBase) was created to address the lack of annotation information and pharmacological profile consideration in existing NR databases.

#### Ligands preparation

The NRLiSt BDB is composed of 9,905 active molecules targeting 27 nuclear receptors (NRs). Active compounds are divided into 2 datasets per target according to their agonist or antagonist profile. All active compounds were extracted from the ChEMBL database and included in the NRLiSt after a manual inspection of the corresponding ligands bioactivity data in the original papers. All inverse-agonists, modulators, agonists/antagonists, weak to partial agonists, weak to partial antagonists and ligands with unknown pharmacological profile were discarded.

In addition 339 human holo structures extracted from the PDB are provided, among which 266 are agonists-bound, 17 are antagonists-bound and 56 are others-bound. Valid active compounds extracted from literature were clustered using chemical fingerprints, and a Tanimoto cut-off of 0.5.

#### Decoys selection

In total 458,981 decoys generated with the DUD-E online tool were provided, with a mean ratio of 1/51 for each dataset.

In further studies, Lagarde et al. integrated the anti-pharmacological profile ligands in the decoy set to orient the screening toward the desired pharmacological profile (Lagarde et al., 2014b). For instance, antagonists were considered as the decoy compounds set for agonists screening research, while agonists were considered as the decoy compounds set for antagonists screening research. In agreement, the corresponding agonist- and antagonist-bound structures were used for SBVS, when available. Results showed that the enrichment is better when the pharmacological profile is considered prior to screening and should therefore be systematically considered to avoid artificially bad ligands enrichment.

### Maximal unbiased benchmarking data sets for HDACs (MUBD-HDACs)

So far, most of the decoy datasets [such as DUD-E (Mysinger et al., 2012) and DEKOIS (Vogel et al., 2011; Bauer et al., 2013)] or decoys generator [such as DecoyFinder (Cereto-Massagué et al., 2012) or the DUD-E generator server] are designed for SBVS purpose. Few databases [i.e., MUV (Rohrer and Baumann, 2009), NRLiSt BDB] are intended to propose benchmarking datasets for LBVS. Xia et al. thus proposed a workflow to fulfill this need, and built up decoy datasets for LBVS targeting the histone deacetylases protein family (HDACs).

#### Ligands preparation

Active compounds were retrieved from the ChEMBL18 database (Gaulton et al., 2012), among molecules annotated with quantitative data (i.e., IC50), manually checked, and filtered (exclusion of salts, molecules with more than 20 rotatable bonds or with a MW of 600 or more). Finally, ligands displaying a Tanimoto coefficient greater than 0.75 based on MACCS fingerprints were removed to exclude analog molecules, and 6 physicochemical properties (MW, logP, HBAs, HBDs, RBs and net Formal Charge–nFC) were computed for all HDACs inhibitors (HDACIs).

#### Decoys selection

The “All-Purchasable Molecules” subset of the ZINC database was used as the initial set of molecules before a two-step filtering:

1. Compounds outside of the bounds of the HDACIs physicochemical properties were removed, as well as molecules with a Tanimoto coefficient (“similarity in structure” or *sims*) greater than 0.75 to any active compounds to circumvent the introduction of potential active structures (false negatives) into the decoy set.
2. To retain only 39 decoys per HDACI, compounds were further filtered to ensure similar physicochemical properties and a random spatial distribution of the decoys around the ligands. A specific metric was assigned to each step, specifically the *simp* (“similarity in properties”) and the *simsdiff* (“*sims* difference”). The *simp* is the Euclidian distance of the physicochemical properties between a target compound and a reference compound. The *simsdiff* between a potential decoy and a query ligand is the average difference between (a) the topological similarity *sims* between the potential decoy and the remaining ligands and (b) the topological similarity *sims* between the query ligands and the remaining ligands. First, a cut-off is applied on the *simp* to ensure properties similarity between ligands and decoy compounds and second, the 39 lowest *simsdiff* decoys for each ligand are selected.

Last, for each ligand, the PDB (Berman et al., 2000) structures of the targeted HDAC isoform were prepared and provided for SBVS data sets. Unlike DUD-E (Mysinger et al., 2012), only Homo sapiens 3D-data were considered.

The MUBD-HDAC datasets for HDAC2 and HDAC8 isoforms were compared to DUD-E (Mysinger et al., 2012) and DEKOIS 2.0 (Ibrahim et al., 2015a) datasets, in terms of structural diversity [Bemis-Murcko atomic frameworks (Bemis and Murcko, 1996)], property matching and ligand enrichment in SB- and LB-VS approaches. The MUBD-HDAC displayed similar to better results in terms of structural diversity and property matching and was more challenging as measured by ligand enrichment using GOLD (Jones et al., 1997) or fingerprints similarity search, in agreement with a higher structural similarity. Finally, the MUBD-HDACs sets displayed small to great improvement in terms of nearer ligands bias (i.e., ligands that are more similar structurally to a ligand than to any decoy), compared to DUD-E and DEKOIS 2.0, respectively. This bias is known to produce artificially positive LBVS evaluation outcomes (Cleves and Jain, 2008) and thus, should be minimized.

Of note, a similar work was done (Xia et al., 2014) on GPCRs using the GLL/GDD database (Gatica and Cavasotto, 2012) as ligands set, and also resulted in reduced artificial enrichment and analog bias compared to the original GLL/GDD sets.

## Discussion and recommendations

### Ideal benchmarking database

The ideal VS benchmarking datasets composition should mimic real-life cases, where a small number of diverse active ligands is embedded into a much larger fraction of inactive compounds. Moreover, both sets of molecules are usually indistinguishable using simple descriptors like their physicochemical properties and share common fragments or functional chemical groups; such features should therefore be transposed to benchmarking datasets design, so that the putative inactive compounds constitute good “decoy” compounds in line with the active compounds and ensure a robust evaluation of the VS methods(Good and Oprea, 2008; Lagarde et al., 2015; Xia et al., 2015).

### Comparison of decoys selection methods for SBVS

Among the recent tools to help create benchmarking sets (MUV, DEKOIS, DUD-E, and MUBD), the main difference resides in the strategy used to achieve their respective objectives: the DUD-E and DEKOIS data sets are designed for evaluating SBVS methods while MUV and MUBD are conceived for benchmarking LBVS approaches. Following this basic distinction, the respective algorithms to generate decoy datasets differ significantly. In the former case, the topological dissimilarity between ligand compounds and decoy compounds is maximized to avoid inclusion of active compounds into decoy datasets. In the latter case, the proper embedding of decoy compounds into the ligands chemical space is of primary importance.

For the DUD-E, the final decoys were randomly selected from the 25% most topologically dissimilar molecules compared to the ligands to ensure unbiased selection of decoy compounds. However, several studies pointed out that bias are still present into DUD-E data sets. For instance, Chaput et al. recently evidenced that the performance of four VS programs (Glide, Gold, FlexX and Surflex) is biased (over-estimated) using the DUD-E. Good performance (as measure by BEDROC curves) could be achieved for all programs when original DUD-E datasets were used, while only Glide was considered successful when chemical library biases (i.e., datasets whose decoys and active compounds differ for nine physicochemical properties) were removed. While the DUD-E was successfully used for numerous studies, this observation clearly showed that there is still place for improvements.

Boeckler's group proposed a similar workflow in DEKOIS and DEKOIS 2.0. A physicochemical similarity over eight properties (and represented by the physicochemical similarity score PSS) is used and the topological dissimilarity between the active compounds and the future decoy compounds is computed as in the DUD-E. However, two main differences have to be noted: (1) the topological dissimilarity was computed using the more elaborated weighted LADS score rather than a 2D fingerprint based Tanimoto coefficient filter and (2) the LADS score was combined with the PSS prior to final selection of the decoys. Therefore, the final decoys selection was balanced by both parameters (physicochemical similarity and topological dissimilarity) rather than using successive arbitrary (even if widely used) thresholds, and was successfully used by Hamza et al. (2014) for drug repurposing. This balance may come at a cost, as evidenced by Xia et al.: DEKOIS datasets for HDAC2 and HDAC8 were shown to be less efficient in terms of property matching between the active compounds and the decoy compounds (Xia et al., 2015). However, the DUD-E and DEKOIS sets perform similarly in enrichment using Gold and DEKOIS perform significantly worse than DUD-E using 2D based similarity search approaches.

### Comparison of decoys selection methods for LBVS

Both DUD-E and DEKOIS databases share the same overall decoy selection procedure by combining topological dissimilarity and physicochemical properties similarity. While adapted to SBVS, this approach may hinder the objective evaluation of LBVS that is very sensitive to topological difference between active and decoy compounds. The MUV datasets (Rohrer and Baumann, 2009) was designed to overcome this specific weakness of the benchmarking datasets. The authors introduced the notion that decoy compounds and active compounds should be homogeneously spread in the chemical space rather than decoy compounds should be topologically dissimilar to the active compounds (as in the DUD-E for instance). The authors tested 18 datasets and claimed that MUV benchmarking datasets displayed neither analogous bias nor artificial enrichment. Furthermore, they noticed that their data sets were SBVS compliant and compared advantageously to the biased DUD sets, leading to a potential broader use of their sets. MUV sets were applied to the evaluation of VS tools (Tiikkainen et al., 2009; Abdo et al., 2010), the training of new QSAR models (Marchese Robinson et al., 2017) or molecular graph convolutions (Kearnes et al., 2016).

As highlighted by Xia et al. “MUV is restricted by the sufficient experimental decoys (chemical space of decoys)” (Xia et al., 2015). Indeed, MUV relies on the availability of experimental data and is restricted to well-studied targets. The authors subsequently proposed the Maximum Unbiased Benchmarking Data sets (MUBD, see section Benchmarking Databases) that was applied to GPCRs (Xia et al., 2014), HDACs (Xia et al., 2015; Hu et al., 2017) and Toll-like receptor 8 (Pei et al., 2015). The MUBD-DecoyMaker algorithm relies on both a minimal and required topological dissimilarity (*sims*) between decoy and active compounds, but makes use of an additional criterion that minimizes the *simsdiff* parameter, i.e., ensures that decoy and active compounds are as similar as possible.

One should note that this additional step (the decoy-actives similarity check) yield datasets also suitable for SBVS; they seemed even more challenging in SBVS (for HDAC2 and HDAC8) as they provided datasets with higher structural similarity (Xia et al., 2015). Thus, these approaches are particularly appealing as they provide benchmarking datasets that (1) are adapted to LB and SB-VS approaches, (2) subsequently allow comparative evaluations of the performance of LB and SB-VS approaches, and (3) may be more challenging for SBVS.

### Fine-tuned benchmarking datasets

The quality of an evaluation lies in the consistency between the retrospectively screened benchmarking datasets and the prospectively screened compound collections as well as the target binding site properties (Ben Nasr et al., 2013). The recent trend to publish protein family-specific datasets or user-provided active compounds dependent decoys generation tools paves the way for a valuable and systematic use of benchmarking datasets prior to prospective VS of large compound collections.

In SBVS, tuned datasets should be used to identify the protocol, conformational sampling, and/or scoring methods that induces the best enrichment in active compounds (Allen et al., 2015, 2017; Lacroix et al., 2016; Li et al., 2016; Nunes et al., 2016; Meirson et al., 2017). For instance, Allen et al. (2015, 2017) evaluated different scoring schemes using DUD-E generated decoys and successfully identified dual EFGR/BRD4 inhibitors. In LBVS, the choice of the dataset is crucial to build a reliable model that can be used to distinguish active compounds from decoy compounds. For example, Ruggeri et al. (2015) used DUD-E generated decoys to define and optimize pharmacophore models that led to the identification of 2 dual competitive inhibitors of *P. Falciparum* M1 (*Pf* A-M1) and M17 (*Pf* A-M17) aminopeptidases.

Of note, when using automatic decoy datasets generation tools, the provided active compounds should be carefully selected to avoid the previously detailed biases.

### Integration of true inactive compounds

Despite the open-data initiatives that should ease the access to data in the near future, the low documentation about negative data (inactive and/or non-binding) is still an open issue. The inclusion of experimental data in a dataset requires great attention since (1) publicly available databases may present annotation errors that should be manually corrected (Lagarde et al., 2014a), and (2) diversity in the type of value and experimental conditions make some data barely comparable. The selection and the use of negative compounds (inactive and/or non-binding) in the evaluation/development of methods is a delicate step that strongly influences the quality of the resulting model. In agreement with Lagarde et al. (2014a) and Kaserer et al. (2015), we recommend that:

1. Interaction data should be extracted from receptor binding or enzymatic activity assays on isolated or recombinant protein; cell-based assays should be avoided because of the many factors that can influence the outcome of the assay (non-specific binding…).
2. Low binders or high IC50/EC50 should not be included in the active set and could be either classified as “inactive,” as negative data or discarded.
3. Experimental bias should be minimized by (a) considering the measured affinity/activity confidence based on the number of documented repeated assays and/or convergent values in different studies and (b) filtering compounds which measured activity/affinity may be an artifact caused by organic chemicals aggregation in aqueous buffers, off-targets effects, cytotoxic effects or interference with optical detection methods (auto-fluorescence and luciferase inhibition).
4. The origin of the protein used in the assay should be considered, favoring 100% identity with the reference.
5. Attention should be paid to the ligand binding-site, particularly for proteins that possess more than one binding site, and for multiple conformation binding sites.

One should note that the integration of inactive/non-binding compounds comes with new basics for datasets design. This case is particularly challenging since the inactive/non-binding compounds are usually extracted from the same chemical series as the active compounds. In this case, small fragments modification can induce important bioactivity loss or gain, thus, clustering active compounds to guarantee diversity and minimize analogous bias would have no meaning. Since the final objective of using such data is to harshly evaluate ability of VS methods to discriminate active from inactive compounds based on small signals, the proximity between active and inactive compounds within a chemotype should be conserved, as well as the similarity within the active compounds of a chemotype. However the over representation of a given chemotype could hinder the evaluation of VS method by masking the enrichment of low populated chemotypes. We suggest that a work should be made to equally represent chemotypes and/or to weight the resulting ROC curve (Ibrahim et al., 2015b).

## Conclusion

Benchmarking databases are widely used to evaluate virtual screening methods. They are particularly important to compare performance of virtual screening methods and therefore to select appropriate protocol prior to large compounds collections screening, and to estimate the reliability of the results of a screening. The characterization of the weaknesses of the first published databases helped designing improved benchmarking datasets with minimized bias. The rational selection of decoy compounds is particularly important to avoid artificial enrichment in the evaluation of the different methods. The diversification of public datasets gathering both active and decoy compounds for a given protein family, and the publication of online decoys generation tools contributed to the democratization of the use of benchmarking studies to help identifying protocols adapted for the query/target system under study. Nowadays, experimental data are being integrated in the decoy compounds set to look for a specific activity or to identify methods fitted for highly similar binders/non binders discrimination. Experimentally validated decoys selection requires careful attention to minimize experimental biases that may arise.

## Author contributions

All authors listed have made substantial, direct and intellectual contribution to the work, and approved it for publication.

### Conflict of interest statement

The authors declare that the research was conducted in the absence of any commercial or financial relationships that could be construed as a potential conflict of interest.

## Footnotes

^1^MDDR licensed by Molecular Design, Ltd., San Leandro, CA.

^2^Available at: drugdesigndata.org

^3^Available online at: http://pubchem.ncbi.nlm.nih.gov/sources#assay

## References

[B1] Abdo A. Chen B. Mueller C. Salim N. Willett P. (2010). Ligand-based virtual screening using Bayesian networks. J. Chem. Inf. Model. 50, 1012–1020. 10.1021/ci100090p 20504032

[B2] Allen B. K. Mehta S. Ember S. W. Schonbrunn E. Ayad N. Schürer S. C. (2015). Large-scale computational screening identifies first in class multitarget inhibitor of EGFR kinase and BRD4. Sci. Rep. 5:srep16924. 10.1038/srep16924 26596901 PMC4657038

[B3] Allen B. K. Mehta S. Ember S. W. J. Zhu J.-Y. Schönbrunn E. Ayad N. G.. (2017). Identification of a novel class of BRD4 inhibitors by computational screening and binding simulations. ACS Omega 2, 4760–4771. 10.1021/acsomega.7b00553 28884163 PMC5579542

[B4] Auld D. S. Southall N. T. Jadhav A. Johnson R. L. Diller D. J. Simeonov A.. (2008). Characterization of chemical libraries for luciferase inhibitory activity. J. Med. Chem. 51, 2372–2386. 10.1021/jm701302v 18363348

[B5] Bauer M. R. Ibrahim T. M. Vogel S. M. Boeckler F. M. (2013). Evaluation and optimization of virtual screening workflows with DEKOIS 2.0 – a public library of challenging docking benchmark sets. J. Chem. Inf. Model. 53, 1447–1462. 10.1021/ci400115b 23705874

[B6] Bemis G. W. Murcko M. A. (1996). The properties of known drugs. 1. Molecular frameworks. J. Med. Chem. 39, 2887–2893. 10.1021/jm9602928 8709122

[B7] Ben Nasr N. Guillemain H. Lagarde N. Zagury J.-F. Montes M. (2013). Multiple structures for virtual ligand screening: defining binding site properties-based criteria to optimize the selection of the query. J. Chem. Inf. Model. 53, 293–311. 10.1021/ci3004557 23312043

[B8] Benson M. L. Smith R. D. Khazanov N. A. Dimcheff B. Beaver J. Dresslar P.. (2008). Binding MOAD, a high-quality protein-ligand database. Nucleic Acids Res. 36, D674–D678. 10.1093/nar/gkm911 18055497 PMC2238910

[B9] Bento A. P. Gaulton A. Hersey A. Bellis L. J. Chambers J. Davies M.. (2014). The ChEMBL bioactivity database: an update. Nucleic Acids Res. 42, D1083–D1090. 10.1093/nar/gkt1031 24214965 PMC3965067

[B10] Berman H. M. Westbrook J. Feng Z. Gilliland G. Bhat T. N. Weissig H. (2000). The protein data bank. Nucleic Acids Res. 28, 235–242. 10.1093/nar/28.1.235 10592235 PMC102472

[B11] Bissantz C. Bernard P. Hibert M. Rognan D. (2003). Protein-based virtual screening of chemical databases. II. Are homology models of G-Protein Coupled Receptors suitable targets? Proteins 50, 5–25. 10.1002/prot.10237 12471595

[B12] Bissantz C. Folkers G. Rognan D. (2000). Protein-based virtual screening of chemical databases. 1. Evaluation of different docking/scoring combinations. J. Med. Chem. 43, 4759–4767. 10.1021/jm001044l 11123984

[B13] Block P. Sotriffer C. A. Dramburg I. Klebe G. (2006). AffinDB: a freely accessible database of affinities for protein–ligand complexes from the PDB. Nucleic Acids Res. 34, D522–D526. 10.1093/nar/gkj039 16381925 PMC1347402

[B14] Braga R. C. Andrade C. H. (2013). Assessing the performance of 3D pharmacophore models in virtual screening: how good are they? Curr. Top. Med. Chem. 13, 1127–1138. 10.2174/1568026611313090010 23651486

[B15] Brozell S. R. Mukherjee S. Balius T. E. Roe D. R. Case D. A. Rizzo R. C. (2012). Evaluation of DOCK 6 as a pose generation and database enrichment tool. J. Comput. Aided Mol. Des. 26, 749–773. 10.1007/s10822-012-9565-y 22569593 PMC3902891

[B16] Cereto-Massagué A. Guasch L. Valls C. Mulero M. Pujadas G. Garcia-Vallvé S. (2012). DecoyFinder: an easy-to-use python GUI application for building target-specific decoy sets. Bioinformatics 28, 1661–1662. 10.1093/bioinformatics/bts249 22539671

[B17] Clark R. D. Webster-Clark D. J. (2008). Managing bias in ROC curves. J. Comput. Aided Mol. Des. 22, 141–146. 10.1007/s10822-008-9181-z 18256892

[B18] Cleves A. E. Jain A. N. (2008). Effects of inductive bias on computational evaluations of ligand-based modeling and on drug discovery. J. Comput. Aided Mol. Des. 22, 147–159. 10.1007/s10822-007-9150-y 18074107

[B19] Cummings M. D. DesJarlais R. L. Gibbs A. C. Mohan V. Jaeger E. P. (2005). Comparison of automated docking programs as virtual screening tools. J. Med. Chem. 48, 962–976. 10.1021/jm049798d 15715466

[B20] Diller D. J. Li R. (2003). Kinases, homology models, and high throughput docking. J. Med. Chem. 46, 4638–4647. 10.1021/jm020503a 14561083

[B21] Eldridge M. D. Murray C. W. Auton T. R. Paolini G. V. Mee R. P. (1997). Empirical scoring functions: I. The development of a fast empirical scoring function to estimate the binding affinity of ligands in receptor complexes. J. Comput. Aided Mol. Des. 11, 425–445. 10.1023/A:1007996124545 9385547

[B22] Empereur-mot C. Guillemain H. Latouche A. Zagury J.-F. Viallon V. Montes M. (2015). Predictiveness curves in virtual screening. J. Cheminformatics 7:52. 10.1186/s13321-015-0100-8 26539250 PMC4631717

[B23] Gatica E. A. Cavasotto C. N. (2012). Ligand and decoy sets for docking to G protein-coupled receptors. J. Chem. Inf. Model. 52, 1–6. 10.1021/ci200412p 22168315

[B24] Gaulton A. Bellis L. J. Bento A. P. Chambers J. Davies M. Hersey A.. (2012). ChEMBL: a large-scale bioactivity database for drug discovery. Nucleic Acids Res. 40, D1100–D1107. 10.1093/nar/gkr777 21948594 PMC3245175

[B25] Good A. C. Oprea T. I. (2008). Optimization of CAMD techniques 3. Virtual screening enrichment studies: a help or hindrance in tool selection? J. Comput. Aided Mol. Des. 22, 169–178. 10.1007/s10822-007-9167-2 18188508

[B26] Guasch L. Sala E. Castell-Auví A. Cedó L. Liedl K. R. Wolber G.. (2012). Identification of PPARgamma partial agonists of natural origin (I): development of a virtual screening procedure and *in vitro* validation. PLoS ONE 7:e50816. 10.1371/journal.pone.0050816 23226391 PMC3511273

[B27] Hamza A. Wagner J. M. Wei N.-N. Kwiatkowski S. Zhan C.-G. Watt D. S.. (2014). Application of the 4D fingerprint method with a robust scoring function for scaffold-hopping and drug repurposing strategies. J. Chem. Inf. Model. 54, 2834–2845. 10.1021/ci5003872 25229183 PMC4210175

[B28] Hawkins P. C. Warren G. L. Skillman A. G. Nicholls A. (2008). How to do an evaluation: pitfalls and traps. J. Comput. Aided Mol. Des. 22, 179–190. 10.1007/s10822-007-9166-3 18217218 PMC2270916

[B29] Hu H. Xia J. Wang D. Wang X. S. Wu S. (2017). A thoroughly validated virtual screening strategy for discovery of novel HDAC3 inhibitors. Int. J. Mol. Sci. 18, 137. 10.3390/ijms18010137 28106794 PMC5297770

[B30] Huang N. Shoichet B. K. Irwin J. J. (2006). Benchmarking sets for molecular docking. J. Med. Chem. 49, 6789–6801. 10.1021/jm0608356 17154509 PMC3383317

[B31] Ibrahim T. M. Bauer M. R. Boeckler F. M. (2015a). Applying DEKOIS 2.0 in structure-based virtual screening to probe the impact of preparation procedures and score normalization. J. Cheminformatics 7:21. 10.1186/s13321-015-0074-6 26034510 PMC4450982

[B32] Ibrahim T. M. Bauer M. R. Dörr A. Veyisoglu E. Boeckler F. M. (2015b). pROC-Chemotype plots enhance the interpretability of benchmarking results in structure-based virtual screening. J. Chem. Inf. Model. 55, 2297–2307. 10.1021/acs.jcim.5b00475 26434782

[B33] Irwin J. J. (2008). Community benchmarks for virtual screening. J. Comput. Aided Mol. Des. 22, 193–199. 10.1007/s10822-008-9189-4 18273555

[B34] Irwin J. J. Shoichet B. K. (2005). ZINC–a free database of commercially available compounds for virtual screening. J. Chem. Inf. Model. 45, 177–182. 10.1021/ci049714+ 15667143 PMC1360656

[B35] Jain A. N. Nicholls A. (2008). Recommendations for evaluation of computational methods. J. Comput. Aided Mol. Des. 22, 133–139. 10.1007/s10822-008-9196-5 18338228 PMC2311385

[B36] Jones G. Willett P. Glen R. C. Leach A. R. Taylor R. (1997). Development and validation of a genetic algorithm for flexible docking. J. Mol. Biol. 267, 727–748. 10.1006/jmbi.1996.0897 9126849

[B37] Kaserer T. Beck K. R. Akram M. Odermatt A. Schuster D. (2015). Pharmacophore models and pharmacophore-based virtual screening: concepts and applications exemplified on hydroxysteroid dehydrogenases. Mol. Basel Switz. 20, 22799–22832. 10.3390/molecules201219880 26703541 PMC6332202

[B38] Kavlock R. Chandler K. Houck K. Hunter S. Judson R. Kleinstreuer N.. (2012). Update on EPA's ToxCast program: providing high throughput decision support tools for chemical risk management. Chem. Res. Toxicol. 25, 1287–1302. 10.1021/tx3000939 22519603

[B39] Kearnes S. McCloskey K. Berndl M. Pande V. Riley P. (2016). Molecular graph convolutions: moving beyond fingerprints. J. Comput. Aided Mol. Des. 30, 595–608. 10.1007/s10822-016-9938-8 27558503 PMC5028207

[B40] Kellenberger E. Rodrigo J. Muller P. Rognan D. (2004). Comparative evaluation of eight docking tools for docking and virtual screening accuracy. Proteins 57, 225–242. 10.1002/prot.20149 15340911

[B41] Kuntz I. D. Blaney J. M. Oatley S. J. Langridge R. Ferrin T. E. (1982). A geometric approach to macromolecule-ligand interactions. J. Mol. Biol. 161, 269–288. 10.1016/0022-2836(82)90153-X 7154081

[B42] Lacroix C. Fish I. Torosyan H. Parathaman P. Irwin J. J. Shoichet B. K.. (2016). Identification of novel smoothened ligands using structure-based docking. PLoS ONE 11:e0160365. 10.1371/journal.pone.0160365 27490099 PMC4973902

[B43] Lagarde N. Ben Nasr N. Jérémie A. Guillemain H. Laville V. Labib T.. (2014a). NRLiSt BDB, the manually curated nuclear receptors ligands and structures benchmarking database. J. Med. Chem. 57, 3117–3125. 10.1021/jm500132p 24666037

[B44] Lagarde N. Delahaye S. Jérémie A. Ben Nasr N. Guillemain H. Empereur-Mot C.. (2017). Discriminating agonist from antagonist ligands of the nuclear receptors using different chemoinformatics approaches. Mol. Inform. 36:1700020. 10.1002/minf.201700020 28671755

[B45] Lagarde N. Delahaye S. Zagury J.-F. Montes M. (2016). Discriminating agonist and antagonist ligands of the nuclear receptors using 3D-pharmacophores. J. Cheminformatics 8:43. 10.1186/s13321-016-0154-2 27602059 PMC5011875

[B46] Lagarde N. Zagury J.-F. Montes M. (2014b). Importance of the pharmacological profile of the bound ligand in enrichment on nuclear receptors: toward the use of experimentally validated decoy ligands. J. Chem. Inf. Model. 54, 2915–2944. 10.1021/ci500305c 25250508

[B47] Lagarde N. Zagury J.-F. Montes M. (2015). Benchmarking data sets for the evaluation of virtual ligand screening methods: review and perspectives. J. Chem. Inf. Model. 55, 1297–1307. 10.1021/acs.jcim.5b00090 26038804

[B48] Li J. Wang H. Li J. Bao J. Wu C. (2016). Discovery of a potential HER2 inhibitor from natural products for the treatment of HER2-positive breast cancer. Int. J. Mol. Sci. 17:1055. 10.3390/ijms17071055 27376283 PMC4964431

[B49] Lionta E. Spyrou G. Vassilatis D. K. Cournia Z. (2014). Structure-based virtual screening for drug discovery: principles, applications and recent advances. Curr. Top. Med. Chem. 14, 1923–1938. 10.2174/1568026614666140929124445 25262799 PMC4443793

[B50] Lorber D. M. Shoichet B. K. (2005). hierarchical docking of databases of multiple ligand conformations. Curr. Top. Med. Chem. 5, 739–749. 10.2174/1568026054637683 16101414 PMC1364474

[B51] Löwer M. Geppert T. Schneider P. Hoy B. Wessler S. Schneider G. (2011). Inhibitors of helicobacter pylori protease HtrA found by ‘virtual ligand’ screening combat bacterial invasion of epithelia. PLoS ONE 6:e17986. 10.1371/journal.pone.0017986 21483848 PMC3069028

[B52] Marchese Robinson R. L. Palczewska A. Palczewski J. Kidley N. (2017). Comparison of the predictive performance and interpretability of random forest and linear models on benchmark data sets. J. Chem. Inf. Model. 57, 1773–1792. 10.1021/acs.jcim.6b00753 28715209

[B53] May A. Zacharias M. (2005). Accounting for global protein deformability during protein-protein and protein-ligand docking. Biochim. Biophys. Acta 1754, 225–231. 10.1016/j.bbapap.2005.07.045 16214429

[B54] McGaughey G. B. Sheridan R. P. Bayly C. I. Culberson J. C. Kreatsoulas C. Lindsley S.. (2007). Comparison of topological, shape, and docking methods in virtual screening. J. Chem. Inf. Model. 47, 1504–1519. 10.1021/ci700052x 17591764

[B55] McGovern S. L. Shoichet B. K. (2003). Information decay in molecular docking screens against holo, apo, and modeled conformations of enzymes. J. Med. Chem. 46, 2895–2907. 10.1021/jm0300330 12825931

[B56] Meirson T. Samson A. O. Gil-Henn H. (2017). An *in silico* high-throughput screen identifies potential selective inhibitors for the non-receptor tyrosine kinase Pyk2. Drug Des. Devel. Ther. 11, 1535–1557. 10.2147/DDDT.S136150 28572720 PMC5441678

[B57] Meng E. C. Shoichet B. K. Kuntz I. D. (1992). Automated docking with grid-based energy evaluation. J. Comput. Chem. 13, 505–524. 10.1002/jcc.540130412

[B58] Meyer K. (2007). WOMBAT—A tool for mixed model analyses in quantitative genetics by restricted maximum likelihood (REML). J. Zhejiang Univ. Sci. B 8, 815–821. 10.1631/jzus.2007.B0815 17973343 PMC2064953

[B59] Muegge I. Martin Y. C. (1999). A general and fast scoring function for protein-ligand interactions: a simplified potential approach. J. Med. Chem. 42, 791–804. 10.1021/jm980536j 10072678

[B60] Munk C. Isberg V. Mordalski S. Harpsøe K. Rataj K. Hauser A. S.. (2016). GPCRdb: the G protein-coupled receptor database – an introduction. Br. J. Pharmacol. 173, 2195–2207. 10.1111/bph.13509 27155948 PMC4919580

[B61] Mysinger M. M. Carchia M. Irwin J. J. Shoichet B. K. (2012). Directory of useful decoys, enhanced (DUD-E): better ligands and decoys for better benchmarking. J. Med. Chem. 55, 6582–6594. 10.1021/jm300687e 22716043 PMC3405771

[B62] Mysinger M. M. Shoichet B. K. (2010). Rapid context-dependent ligand desolvation in molecular docking. J. Chem. Inf. Model. 50, 1561–1573. 10.1021/ci100214a 20735049

[B63] Neves M. A. Totrov M. Abagyan R. (2012). Docking and scoring with ICM: the benchmarking results and strategies for improvement. J. Comput. Aided Mol. Des. 26, 675–686. 10.1007/s10822-012-9547-0 22569591 PMC3398187

[B64] Nunes R. R. Costa M. D. Santos B. D. Fonseca A. L. Ferreira L. S. Chagas R. C.. (2016). Successful application of virtual screening and molecular dynamics simulations against antimalarial molecular targets. Mem. Inst. Oswaldo Cruz 111, 721–730. 10.1590/0074-02760160207 27982302 PMC5146734

[B65] Okuno Y. Yang J. Taneishi K. Yabuuchi H. Tsujimoto G. (2006). GLIDA: GPCR-ligand database for chemical genomic drug discovery. Nucleic Acids Res. 34, D673–D677. 10.1093/nar/gkj028 16381956 PMC1347391

[B66] Pei F. Jin H. Zhou X. Xia J. Sun L. Liu Z.. (2015). Enrichment assessment of multiple virtual screening strategies for Toll-like receptor 8 agonists based on a maximal unbiased benchmarking data set. Chem. Biol. Drug Des. 86, 1226–1241. 10.1111/cbdd.12590 26017460

[B67] Placzek S. Schomburg I. Chang A. Jeske L. Ulbrich M. Tillack J.. (2017). BRENDA in 2017: new perspectives and new tools in BRENDA. Nucleic Acids Res. 45, D380–D388. 10.1093/nar/gkw952 27924025 PMC5210646

[B68] Rarey M. Kramer B. Lengauer T. Klebe G. (1996). A fast flexible docking method using an incremental construction algorithm. J. Mol. Biol. 261, 470–489. 10.1006/jmbi.1996.0477 8780787

[B69] Repasky M. P. Murphy R. B. Banks J. L. Greenwood J. R. Tubert-Brohman I. Bhat S.. (2012). Docking performance of the glide program as evaluated on the Astex and DUD datasets: a complete set of glide SP results and selected results for a new scoring function integrating WaterMap and glide. J. Comput. Aided Mol. Des. 26, 787–799. 10.1007/s10822-012-9575-9 22576241

[B70] Rognan D. Lauemoller S. L. Holm A. Buus S. Tschinke V. (1999). Predicting binding affinities of protein ligands from three-dimensional models: application to peptide binding to class I major histocompatibility proteins. J. Med. Chem. 42, 4650–4658. 10.1021/jm9910775 10579827

[B71] Rohrer S. G. Baumann K. (2009). Maximum unbiased validation (MUV) data sets for virtual screening based on PubChem bioactivity data. J. Chem. Inf. Model. 49, 169–184. 10.1021/ci8002649 19434821

[B72] Roth B. L. Lopez E. Patel S. Kroeze W. K. (2000). The multiplicity of serotonin receptors: Uselessly diverse molecules or an embarrassment of riches? Neuroscientist 6, 252–262. 10.1177/107385840000600408

[B73] Ruggeri C. Drinkwater N. Sivaraman K. K. Bamert R. S. McGowan S. Paiardini A. (2015). Identification and validation of a potent dual inhibitor of the *P. falciparum* M1 and M17 aminopeptidases using virtual screening. PLoS ONE 10:e0138957. 10.1371/journal.pone.0138957 26406322 PMC4583420

[B74] Simeonov A. Jadhav A. Thomas C. J. Wang Y. Huang R. Southall N. T.. (2008). Fluorescence spectroscopic profiling of compound libraries. J. Med. Chem. 51, 2363–2371. 10.1021/jm701301m 18363325

[B75] Sliwoski G. Kothiwale S. Meiler J. Lowe E. W. (2014). Computational methods in drug discovery. Pharmacol. Rev. 66, 334–395. 10.1124/pr.112.007336 24381236 PMC3880464

[B76] Southan C. Sharman J. L. Benson H. E. Faccenda E. Pawson A. J. Alexander S. P.. (2016). The IUPHAR/BPS guide to PHARMACOLOGY in 2016: towards curated quantitative interactions between 1300 protein targets and 6000 ligands. Nucleic Acids Res. 44, D1054–D1068. 10.1093/nar/gkv1037 26464438 PMC4702778

[B77] Spitzer R. Jain A. N. (2012). Surflex-dock: docking benchmarks and real-world application. J. Comput. Aided Mol. Des. 26, 687–699. 10.1007/s10822-011-9533-y 22569590 PMC3398190

[B78] Stumpfe D. Bajorath J. (2011). Applied virtual screening: strategies, recommendations, and caveats, in Virtual Screening: Principles, Challenges, and Practical Guidelines, ed Sotriffer C. (Weinheim: Wiley-VCH Verlag GmbH and Co. KGaA), 291–318. 10.1002/9783527633326.ch11

[B79] Tanrikulu Y. Krüger B. Proschak E. (2013). The holistic integration of virtual screening in drug discovery. Drug Discov. Today 18, 358–364. 10.1016/j.drudis.2013.01.007 23340112

[B80] Tiikkainen P. Markt P. Wolber G. Kirchmair J. Distinto S. Poso A.. (2009). Critical comparison of virtual screening methods against the MUV data set. J. Chem. Inf. Model. 49, 2168–2178. 10.1021/ci900249b 19799417

[B81] Triballeau N. Acher F. Brabet I. Pin J.-P. Bertrand H.-O. (2005). Virtual screening workflow development guided by the “receiver operating characteristic” curve approach. Application to high-throughput docking on metabotropic glutamate receptor subtype 4. J. Med. Chem. 48, 2534–2547. 10.1021/jm049092j 15801843

[B82] Verdonk M. L. Berdini V. Hartshorn M. J. Mooij W. T. Murray C. W. Taylor R. D.. (2004). Virtual screening using protein-ligand docking: avoiding artificial enrichment. J. Chem. Inf. Comput. Sci. 44, 793–806. 10.1021/ci034289q 15154744

[B83] Vilar S. Karpiak J. Costanzi S. (2010). Ligand and structure-based models for the prediction of ligand-receptor affinities and virtual screenings: development and application to the β2-adrenergic receptor. J. Comput. Chem. 31, 707–720. 10.1002/jcc.21346 19569204 PMC2818076

[B84] Vogel S. M. Bauer M. R. Boeckler F. M. (2011). DEKOIS: Demanding evaluation kits for objective *in silico* screening—a versatile tool for benchmarking docking programs and scoring functions. J. Chem. Inf. Model. 51, 2650–2665. 10.1021/ci2001549 21774552

[B85] von Korff M. Freyss J. Sander T. (2009). Comparison of ligand- and structure-based virtual screening on the DUD data set. J. Chem. Inf. Model. 49, 209–231. 10.1021/ci800303k 19434824

[B86] Wallach I. Lilien R. (2011). Virtual decoy sets for molecular docking benchmarks. J. Chem. Inf. Model. 51, 196–202. 10.1021/ci100374f 21207928

[B87] Wang R. Fang X. Lu Y. Wang S. (2004). The PDBbind database: collection of binding affinities for protein-ligand complexes with known three-dimensional structures. J. Med. Chem. 47, 2977–2980. 10.1021/jm030580l 15163179

[B88] Wang R. Fang X. Lu Y. Yang C.-Y. Wang S. (2005). The PDBbind database: methodologies and updates. J. Med. Chem. 48, 4111–4119. 10.1021/jm048957q 15943484

[B89] Wang R. Liu L. Lai L. Tang Y. (1998). SCORE: a new empirical method for estimating the binding affinity of a protein-ligand complex. Mol. Model. Annu. 4, 379–394. 10.1007/s008940050096

[B90] Wang Y. Bryant S. H. Cheng T. Wang J. Gindulyte A. Shoemaker B. A.. (2017). PubChem BioAssay: 2017 update. Nucleic Acids Res. 45, D955–D963. 10.1093/nar/gkw1118 27899599 PMC5210581

[B91] Warren G. L. Andrews C. W. Capelli A.-M. Clarke B. LaLonde J. Lambert M. H.. (2006). A critical assessment of docking programs and scoring functions. J. Med. Chem. 49, 5912–5931. 10.1021/jm050362n 17004707

[B92] Wei B. Q. Baase W. A. Weaver L. H. Matthews B. W. Shoichet B. K. (2002). A model binding site for testing scoring functions in molecular docking. J. Mol. Biol. 322, 339–355. 10.1016/S0022-2836(02)00777-5 12217695

[B93] Wishart D. S. Knox C. Guo A. C. Cheng D. Shrivastava S. Tzur D.. (2008). DrugBank: a knowledgebase for drugs, drug actions and drug targets. Nucleic Acids Res. 36, D901–D906. 10.1093/nar/gkm958 18048412 PMC2238889

[B94] Xia J. Jin H. Liu Z. Zhang L. Wang X. S. (2014). An unbiased method to build benchmarking sets for ligand-based virtual screening and its application to GPCRs. J. Chem. Inf. Model. 54, 1433–1450. 10.1021/ci500062f 24749745 PMC4038372

[B95] Xia J. Tilahun E. L. Kebede E. H. Reid T.-E. Zhang L. Wang X. S. (2015). Comparative modeling and benchmarking data sets for human histone deacetylases and sirtuin families. J. Chem. Inf. Model. 55, 374–388. 10.1021/ci5005515 25633490 PMC4677826

